# Use of miltefosine to treat canine visceral leishmaniasis caused by *Leishmania infantum* in Brazil

**DOI:** 10.1186/s13071-019-3323-0

**Published:** 2019-02-08

**Authors:** Fabio dos Santos Nogueira, Valdir Carlos Avino, Fredy Galvis-Ovallos, Vera Lucia Pereira-Chioccola, Marcio Antonio Batistella Moreira, Ana Paula Peres Lopes Romariz, Leticia M. Molla, Ingrid Menz

**Affiliations:** 1 Fundação Educacional de Andradina, São Paulo, Andradina Brazil; 2 Departamento de Assuntos Regulatórios e Desenvolvimento da Virbac Brasil, São Paulo, Brazil; 3 Programa de Pós-Graduação em Saúde Pública, Faculdade de Saúde Pública, Universidade São Paulo, São Paulo, Brazil; 4 Laboratório de Biologia Molecular de Parasitas e Fungos, Instituto Adolfo Lutz, São Paulo, Brazil; 5 Faculdade de Medicina Veterinária, Universidade Anhembi Morumbi, São Paulo, Brazil; 6 Ingrid Menz Self-employed Veterinarian, São Paulo, Campinas Brazil

**Keywords:** Canine visceral leishmaniasis, Miltefosine, Clinical signs, qPCR, Infectiveness, Xenodiagnosis, Treatment

## Abstract

**Background:**

Visceral leishmaniasis (VL) is an infectious disease with a variety of clinical signs. The main form of parasite transmission to humans and other mammalian hosts is through the bite of infected arthropod females with *Lutzomyia longipalpis* serving as the main vector in the Americas. Dogs are the main urban domestic reservoirs of the parasite and the main source of vector infection due to their high prevalence in endemic areas and the large number of parasites in the skin of infected animals. Although miltefosine has been used in Europe since 2002 for treatment of VL infected dogs, in the Americas the treatment of dogs has not been recommended. Therefore, this study aimed to evaluate efficacy of miltefosine observing a reduction of clinical signs in infected dogs and the infectiveness to the vector by *Leishmania* (*L.*) *infantum*.

**Methods:**

To our knowledge, this is the first controlled study using qPCR and xenodiagnosis to evaluate the efficacy of miltefosine (Milteforan®, Virbac) as a single treatment in Brazil*.* Thirty-five adult dogs with canine visceral leishmaniasis (CVL), confirmed by clinical and laboratory tests, were included in this study. They received miltefosine at a dose of 2 mg/kg every 24 h for 28 days. The dogs were observed over a three-month period, during which clinical evaluations based on a scoring system were conducted at pre-established times. Parasite load was assessed by cytology and real-time polymerase chain reaction (qPCR). Transmissibility to the vector was evaluated by xenodiagnosis.

**Results:**

At the end of the period, the following were observed: (i) the remission of clinical signs with a reduction in clinical scores for 94.2% of the animals; (ii) a statistically significant reduction (98.7%) in parasitic load by qPCR; and (iii) a reduction in infectivity to sand flies. After treatment, 74.2% of the animals remained or had become non-infectious.

**Conclusions:**

Our study indicates that the use of miltefosine administered orally for 4 weeks contributes to a clinical improvement and reduction in infectivity of dogs to *L. infantum.*

## Background

Visceral leishmaniasis (VL) is an infectious, non-contagious, chronic disease with significant clinical and epidemiological control priority in the world. In the last few decades, epidemiological changes in VL, including increases in incidence and lethality rate and its spread to new and even urban areas, have been observed [1–5].

*Leishmania* (*Leishmania*) *infantum* is the aethiological agent of canine visceral leishmaniasis (CVL) distributed in the Old and New World. The main form of transmission of the parasite to humans and other mammalian hosts is through the bite of infected sand flies (Diptera: Psychodidae). In Brazil, the species involved in the transmission are mainly *Lu. longipalpis* and *Lu. cruzi*. Unlike in European countries, where there are two well-defined transmission seasons, in Brazil, in the areas with an occurrence of *Lutzomyia longipalpis*, this vector can be found throughout the entire year. In general, the population density of sand flies increases with high temperature and high relative humidity, resulting in periods of high risk of transmission of the parasite.

Phlebotomine infection occurs when females bite an infected host, thereby ingesting amastigotes. These amastigotes undergo successive divisions and are progressively transformed into infectious metacyclic promastigotes, which are regurgitated in the skin of mammalian hosts during a new blood meal [6–8].

CVL diagnosis has been challenging for public health professionals due to the existence of asymptomatic dogs, the high variability of clinical signs and the difficulty of achieving a diagnostic with high sensitivity and specificity. However, new methods based on DNA sequencing are being applied to parasite diagnosis. The use of real-time PCR (qPCR) has expanded over the past decades, since it is possible to detect parasite DNA in infected animals regardless of their clinical status. In addition, qPCR is able to quantify parasitic load and monitor follow-up treatment [9].

Xenodiagnosis is a useful method for the identification and isolation of parasites in their natural arthropod vectors, or for identification of the infectiveness of the infected host [10, 11]. This method has been used to evaluate the infectivity of *Lu. longipalpis* females which fed on naturally or experimentally infected dogs and the association between this infectivity and host symptoms [12–15].

Different treatment protocols have shown that parasitic forms in the skin and lymphoid organs of dogs are significantly reduced after treatment. Since most of these treated animals continue to be reservoirs, and consequently a source of infection, their monitoring is of extreme importance [3, 16–18].

Miltefosine (hexadecilfosfocoline) was originally studied and classified as an anti-tumor drug [19] and its leishmanicide potential was identified in the 1980s. Its compound is the first oral anti-*Leishmania* drug, which was studied in partnership with the World Health Organization (WHO) and registered as efficient for treatment of infections caused by *L.* (*L.*) *donovani* in humans [20, 21]. Miltefosine inhibits the biosynthesis of the glycosyl phosphatidyl inositol (GPI) receptor, the key molecule for *Leishmania* intracellular survival. It also interferes with the synthesis of phospholipase and protein kinase C, which are *Leishmania*-specific. The metabolic action of this compound can affect the biosynthesis of glycolipids and membrane glycoproteins of the parasite, causing apoptosis. Other studies suggest that this drug has immunomodulatory properties [22–25].

In 2007, Virbac Laboratories launched miltefosine on the European veterinary market as Milteforan for the purpose of treating dogs with CVL. According to Gradoni et al. [26], treatment of asymptomatic and oligosymptomatic dogs results in high rates of recovery and avoidance of the development of clinical disease. According to the WHO [27], the reduction of cutaneous parasitism and clinical signs, and the recovery of cellular immune response, could reduce the capacity to infect sand flies, consequently reducing the prevalence of the disease in canines and humans in endemic areas [11, 26, 28, 29]. Based on these data, the present study aimed to evaluate the efficacy of miltefosine in reducing clinical signs in naturally infected dogs and the infectivity to sand flies.

## Methods

### Dog housing conditions

This study was conducted in Andradina, located in the northwestern region of the state of São Paulo, Brazil, an area endemic for VL with canine and human transmission [27]. Dogs were maintained in a 45 m^2^ kennel with an antechamber. The kennels were completely protected with a 1 mm mesh tissue screen to prevent sand fly access. As a vector surveillance measure, a CDC light trap was installed and turned on daily between 17:00 and 06:00 h. Video cameras were used to monitor the kennels for the occurrence of dog fights and accidents.

### Dogs

For this study 35 adult dogs (18 males and 17 females), weighing between 4–24 kg and of different breeds (2 Dachshunds, 2 Poodles, 3 Brazilian Terriers, 1 Cocker Spaniel and 27 mixed breeds), were selected. All dogs had previously been naturally infected by *L.* (*L.*) *infantum.* CVL was determined by clinical, serological and molecular diagnoses. All dogs were neutered, spayed, and fed with a balanced commercial dog food *ad libitum*. They were kept inside a screened kennel and were microchipped with Virbac Backhome® microchips and then photographed with their microchip numbers.

### Inclusion, exclusion and efficacy criteria

Dogs showing clinical signs characteristic of CVL and with infection status proven by serological, parasitological and/or molecular diagnosis were selected to start the therapy. Serology was performed using the ELISA method and the immunochromatographic DPP test at Instituto Adolfo Lutz in São Paulo, Brazil. All dogs were positive for *L.* (*L.*) *infantum* (ELISA cut-off = 0.174).

Dogs with significant alterations in renal and/or hepatic function or with other infectious diseases were excluded.

Efficacy criteria were determined by reduction in clinical scores [18, 30, 31], in decreasing of parasite DNA and infectiveness to sand flies. The score was defined according to the severity of each clinical sign and the final value was obtained from the sum of all the values. Quantitative parasite load was estimated through qPCR and the transmissibility of parasites to sand flies was evaluated through xenodiagnosis.

### Sample size

The sample size was determined by using a confidence interval of 95%, aiming for results similar to those found in a study conducted by Virbac in France [Study code: F-107.010000-60003], which showed a treatment success rate of 82.7% and a variability of 70–96%. Thus, the minimum number of dogs was 32. Thirty-five animals were initially selected, taking into account possible losses by mortality or fights. The number of dogs requested for this study was obtained according to the following equation (Lwanga & Lemeshow [32]):

$$ n=\frac{\left\{{\left[z(alpha)\right]}^2\times p\times \left(1-p\right)\right\}}{d^2} $$

where *n* is the sample size; *z*(alpha) is the value obtained through the normal distribution to obtain a confidence interval of 95%: *z*(alpha) = 1.96%; p is the expected ratio; and d is the accuracy of estimate (d = range size/2).

### Experimental design

The dogs were treated orally with miltefosine (Milteforan®, Virbac) 2 mg/kg body weight, for four weeks (W0-W3). After each administration, the dogs were observed for 1 h to monitor for vomiting and/or regurgitation to ensure complete absorption of the drug. Animal weight, infection status (serological, cytological and parasite load-qPCR) and infectivity to sand flies (xenodiagnosis) were evaluated before treatment at week 0 (W0). Dog weight and clinical status were evaluated every two weeks from the beginning of treatment, through W12. Serological, cytological and parasite load-qPCR were evaluated at W6 and W12. Infectivity to sand flies was evaluated again at W12.

### Clinical evaluation

The dogs were weighed and given routine physical examinations immediately before beginning therapy and at 14-day intervals. The same researcher conducted all clinical evaluations in a minimum observation time of 20 min per dog to maintain consistency.

Clinical scores [30] were classified according to the severity of clinical signs as shown in Table 1.

**Table 1:** Clinical scores used to evaluate variables before and after treatment

Clinical signs	Intensity (scores)			
	0	1	2	3
Anorexia	A	P		
Polyuria/polydipsia	A	P		
Epistaxis	A	P		
Splenomegaly	A	P		
Vomiting	A	P		
Digestive disorders	A	P		
Uveitis	A	P		
Keratitis	A	P		
Arthritis/limping	A	P		
Onychogryphosis	A	M	I	
Weight loss	A	M	I	
Conjunctivitis	A	M	I	S
Blepharitis	A	M	I	S
Lymphadenomegaly	A	M	I	S
Ulcers	A	M	I	S
Nodules	A	M	I	S
Peeling	A	M	I	S
Depigmentation	A	M	I	S
Hyperpigmentation	A	M	I	S
Hyperkeratosis (feet)	A	M	I	S
Hyperkeratosis and/or Ulcers in nasal region	A	M	I	S

*Abbreviations*: *A* absent, *P* present, *M* moderate, *I* intense, *S* severe

### Skin qPCR

Skin fragments obtained from the ear pinna, collected at the same site immediately after xenodiagnosis, were analysed for quantification of parasitic load. Prior to DNA extraction, the skin samples were digested in a lysis buffer (10 mM Tris-HCl, pH 8.0; 10 mM EDTA, 0.5% SDS; 0.01% N-laurilsarcozil and 100 g/ml proteinase K) and then incubated in a water bath at 56 °C for 2–18 h until complete tissue lysis had occurred [33]. The DNA molecules were extracted using a QIAamp DNA Mini Kit (Qiagen, Venlo, Netherland) according to the manufacturer’s instructions and in equipment specifically used for DNA purification (a QIAcube robotic workstation, Qiagen). The concentrations and purity of DNA molecules were determined by the optical density (OD) ratio at 260/280 nm in a NanoDrop ND1000 (Thermo Scientific, Waltham, USA). qPCR had been previously standardized at the Instituto Adolfo Lutz (São Paulo) using serial dilutions of DNA extracted from reference strain cultures of *L.* (*L.*) *infantum* (MHOM/BR/1972/LD) [34]. The set of molecular markers used in the qPCR was LinJ31, sense and reverse (5'-CCG CGT GCC TGT CG-3' and 5'-CCC ACA CAA GCG GGA ACT-3'), and a TaqMan probe MGB (5'-CCT CCT TGG ACT TTG C-3'), marked with FAM (region 5') and with NFQ (region 3') [35]. The quality of the extracted DNA was confirmed by amplification of the canine gene glyceraldehyde-3-phosphate dehydrogenase, spermatogenicto (GAPDHS; GenBank: XM_533693.2) (Applied Biosystems, Waltham, USA), which was used as an internal control gene. The reactions were conducted with a final volume of 20 μl. The canine samples (3 μl of 100 ng/μl) or DNA control (50 ng) were added to a mixture of 10 μl of 2× TaqMan Universal PCR Master Mix and 1 μl of the molecular marker mix (18 μM of sense and reverse molecular markers and 5 μM of TaqMan probe). The amplifications were conducted using an Applied Biosystems 7500 Real-time PCR, using a thermal cycle including 2 min at 50 °C and 10 min at 95 °C, followed by 40 cycles of 95 °C for 15 s and 60 °C for 1 min. Each DNA extraction set also included a negative tissue sample for *Leishmania* spp. together with the unknown sample in order to monitor cross-contamination during extraction. In each PCR run, a blank control consisting of DNA-free water plus PCR mix was used as blank control. Separate rooms were used for (i) DNA extraction; (ii) PCR mix and primer preparation; and (iii) addition of DNA from clinical samples (in duplicate) and positive control [34].

The results were based on a standard curve where known concentrations of parasites were used to perform the qPCR [34]. The curve had been constructed using seven different DNA concentrations (in triplicate) extracted from *L.* (*L.*) *infantum* (1 × 10^7^ to 1 × 10^-1^ promastigotes). The cycle threshold (C_t_) values were plotted on a graph (average of triplicates) *versus* the concentrations of DNA to determine the limit of detection of molecular marker LinJ31.

Parasite concentrations (number of amplified copies/3 μl DNA sample) were calculated using the linear regression equation [36]

$$ y= ax+b $$

where *y* is C_t_); *a* is the slope of the curve; *x* is the number of parasites; and *b* is the detection limit, where the curve crosses the y-axis (y-intercept). The detection limit of LinJ31 for *L.* (*L.*) *infantum* was at C_t_ of 37.75 with R2 of 0.9957. Then the number of amplified copies/3 μl DNA sample was log_10_-transformed.

### Xenodiagnosis

To avoid repelling or killing the sand flies, no topical and/or oral treatment against ectoparasites were applied before or during the study. After clinical evaluation, the dogs were sedated intramuscularly with acepromazine 1% (Acepram, Vetnil), 0.22 mg/kg and anesthetized intravenously with tiletamine and zolazepam (Zoletil®50, Virbac), 0.12 ml/kg and transferred to the xenodiagnosis room. The sand fly feeding was conducted in the right internal ear of each dog, and a skin fragment from the same ear was collected for qPCR. This procedure was carried out using a 4 mm diameter “punch” (Dermatological Sterile Disposable Punch, Kolplast, Paulinia, Brazil), sterile surgical anatomical tweezers, a scalpel blade, a needle holder and mononylon thread. The fragments were immediately placed in 1.5 ml tubes (Eppendorf PCR Tubes, Eppendorf®, São Paulo, Brazil) with saline solution 0.9% for the qPCR procedure.

**Fig. 1:**
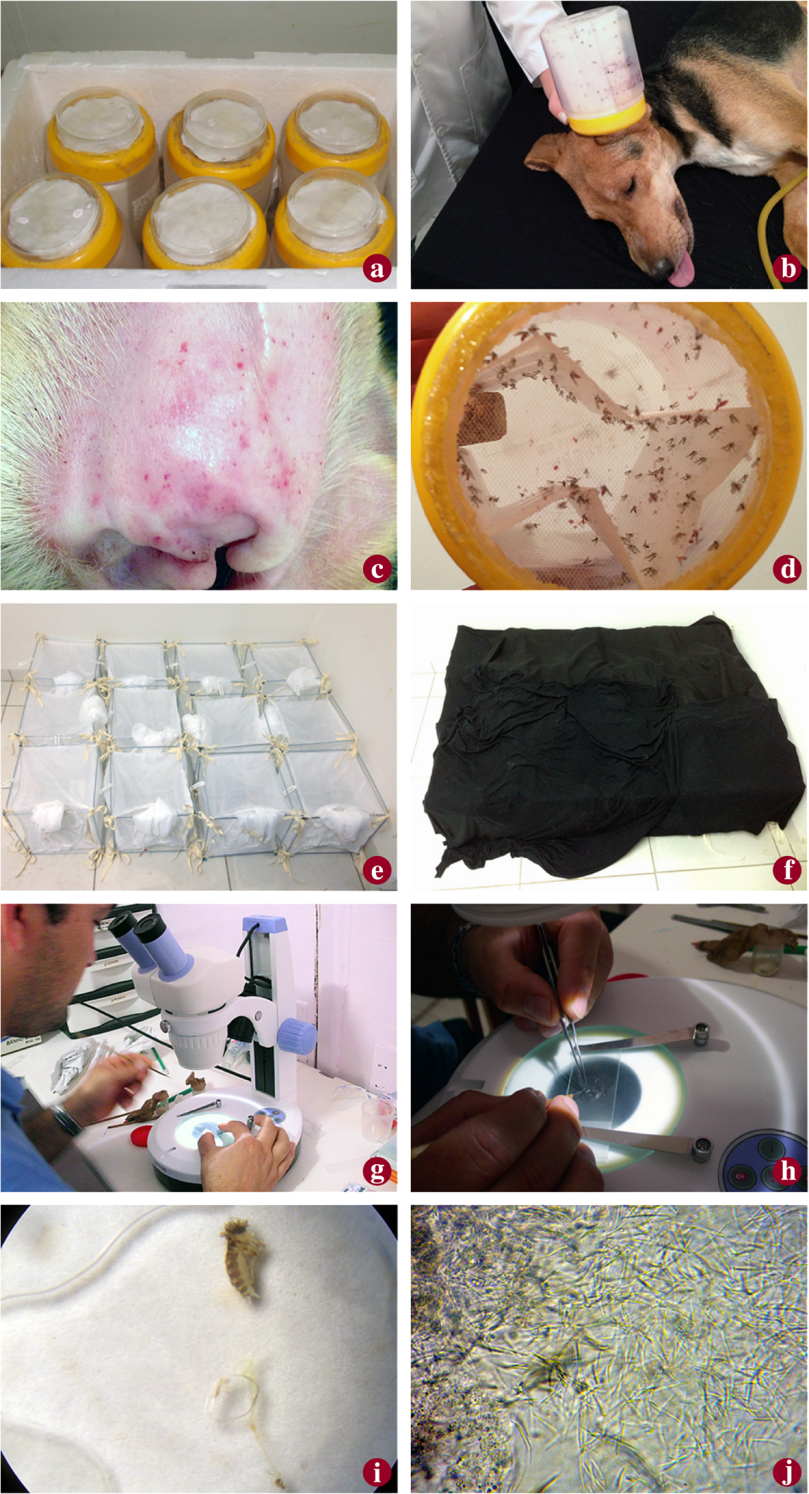
Method of xenodiagnosis, including use of “phlebocontainers” for transportation (**a**), placement of a “phlebocontainer” on the dogs ear (**b**), verification of bites (**c**), verification of engorgement (**d**), containment during transformation of the *Leishmania* in the sand flies (**e**, **f**), phlebotomine dissection (**g**, **h**, **i**) and microscopic visualisation of *Leishmania* (**j**)

*Lutzomyia longipalpis* females used for the xenodiagnosis were from a closed colony kept in the Departamento de Parasitologia of the Universidade Federal de Minas Gerais (UFMG). For transport to the isolated kennel, the insects were maintained in a container designed by da Costa-Val et al. [14] consisting of a transparent box 10 cm high by 8.7 cm in diameter, covered with a nylon screen lid, 10 cm in diameter, 80 rows per cm^2^, fastened to the outer edges of the box with silicon glue (Fig. 1a). Cotton moistened with water and sucrose 10% was placed on top of the screen lid to feed the insects during transport. The “phlebocontainers”, each with 60 to 75 sand flies of which 70% were females, were carefully placed in styrofoam boxes with moistened paper to preserve humidity at 70–80% and transported to the location of the experiment. Due to the aggregated feeding behavior of this species, male insects (30%) were included to stimulate the females during their feeding on the dogs. After transport, the “phlebocontainers” were evaluated for viability of the insects and taken to the xenodiagnosis room to be maintained at an ideal temperature (between 25–28 °C) and humidity levels for 24 h. A container was then placed over one of each dog’s ears, covered with a black cloth, and left in place for 60 min to expose the ear to the sand flies. After exposure the insects were released into individual cages (30 × 30 cm) of the same 1 mm mesh tissue screen, coded with the animal’s microchip number, for stabilization of the peritrophic membrane and maintenance until dissection of the females. A cotton wad soaked with sucrose 10% solution was placed on each cage for 6 days. After this period, the sand flies were anesthetized with ether, transferred to small flasks, and taken to the clinical laboratory for dissection. Parasite count was undertaken as described by Diniz et al. [37]. The number of infected and uninfected females was determined from the total number of dissected females per dog. Information was obtained on the different sites in the gut where the parasites were found. After defecation, the females were dissected and evaluated to detect *L. infantum* promastigotes under an optical microscope at 400× (Zeiss® Cx40, Jena, Germany). The dogs were considered infective if at least one parasite was found in the sand fly. Details about the stages of xenodiagnosis are presented in Fig. 1b-j.

### Data analysis

The non-parametric Wilcoxon test [38] was used for comparison of averages taken at two evaluation time points.

Repeated measures analysis of variance [39] was used to compare weight, clinical score, parasite load by qPCR and infectivity to sand flies. When the assumption of normality of data was rejected, Friedman’s non-parametric test [38] was used. The significance level used for the tests was 5%. SPSS 17.0 software for Windows was used for the calculations.

## Results

### Variation in dog weights

**Fig. 2:**
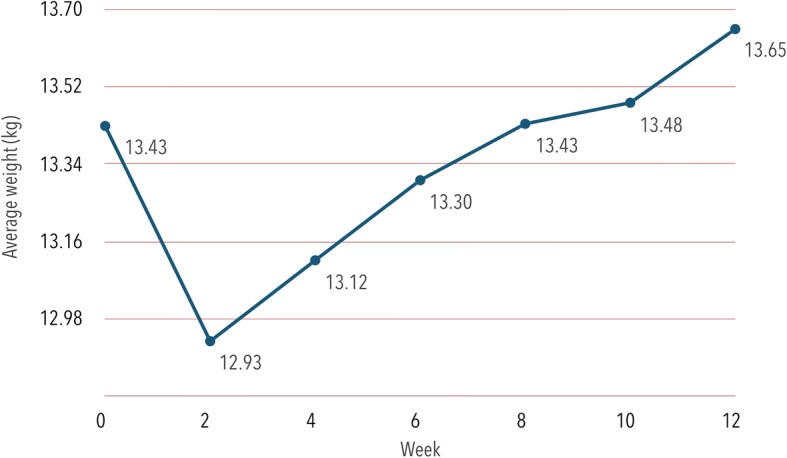
Average dog weight during 12 weeks of observation

During the 12-week period of observation (W0 to W12), the dogs showed a weight gain. Using analysis of variance, a significant change in dog weight throughout the period of evaluation (Repeated measures ANOVA *F*_(6,204)_ = 7.88, *P* < 0.001) was found. There was a significant difference in weight loss between W0 and W2 (Repeated measures ANOVA: *F*_(1,34)_ = 7.85, *P* = 0.008) as well as W0 and W4 (Repeated measures ANOVA contrast W0-W4: *F*_(1,34)_ = 5.52, *P* = 0.025). Significantly lower values were obtained for W4 than for W6 (Repeated measures ANOVA contrast W4-W6: *F*_(1,34)_ = 6.20, *P* = 0.018), W8 (Repeated measures ANOVA contrast W4-W8: *F*_(1,34)_ = 11.93, *P* = 0.002), W10 (Repeated measures ANOVA contrast W4-W10: *F*_(1,34)_ = 14.04, *P* < 0.001) and W12 (Repeated measures ANOVA contrast W4-W12: *F*_(1,34)_ = 17.08, *P* < 0.001) (Fig. 2).

### Clinical evaluation

**Fig. 3:**
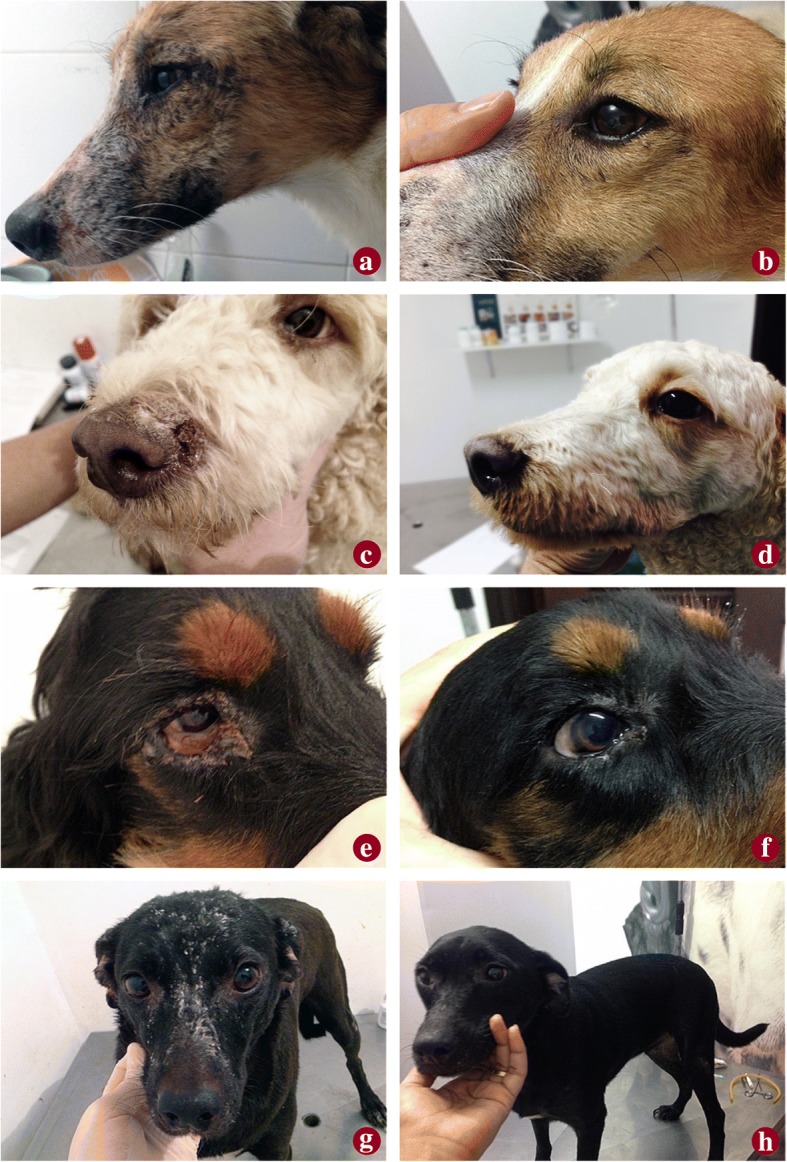
Dogs before (**a**, **c**, **e**, **g**) and 60 days after miltefosine treatment (**b**, **d**, **f**, **h**)

The most frequently observed clinical signs before application of the drug were lymphadenopathy (100%), muscular atrophy (88%), onychogryphosis (74%), blepharitis (74%), localized and/or generalized ulcers (68%), desquamation (60%), alopecia (57%), hyperpigmentation (57%) and cutaneous nodules (40%). As shown in Table 2, the average clinical scores reflected a highly heterogeneous group. Before drug administration, 77.14% (27/35) had clinical scores over 10, considered to be very symptomatic. During the observation periods there was a statistically significant decrease (Repeated measures ANOVA: *F*_(6,204)_ = 69.95, *P* < 0.001), with a progressive reduction in average scores between weeks W0 (16.29 ± 7.57) and W2 (15.26 ± 7.45), W4 (12.14 ± 5.31), W6 (9.26 ± 4.45), W8 (7.23 ± 3.83), W10 (5.46 ± 3.08) and W12 (5.17 ± 3.12). Dogs showed a reduction in average score from 16.29 to 5.17. Figure 3 shows dogs before treatment (Fig. 3a, c, e, g) and 60 days after the end of treatment (W12) (Fig. 3b, d, f, h). One dog (#8) with a score of five at W0 and few clinical signs did not show a reduction, but rather an increase at W12 (score of 8).

**Table 2:** Percent reduction in clinical scores during treatment

Dog ID	Score			% reduction W0-W12
	W0	W6	W12	
1	17	12	5	70.59
2	19	15	10	47.37
3	17	6	4	76.47
4	17	12	4	76.47
5	14	7	4	71.43
6	11	7	3	72.73
7	21	4	5	76.19
8	5	9	8	-60.00
9	13	13	4	69.23
10	27	14	6	77.78
11	7	3	5	28.57
12	15	8	2	86.67
13	14	4	6	57.14
14	16	11	4	75.00
15	24	16	7	70.83
16	17	9	6	64.71
17	21	11	5	76.19
18	21	11	3	85.71
19	11	5	2	81.82
20	21	11	8	61.90
21	13	6	4	69.23
22	27	21	15	44.44
23	9	5	3	66.67
24	4	4	4	0.00
25	23	10	13	43.48
26	27	14	3	88.89
27	8	8	2	75.00
28	30	13	9	70.00
29	11	7	1	90.91
30	31	17	7	77.42
31	10	8	3	70.00
32	9	1	2	77.78
33	3	4	2	33.33
34	11	8	4	63.64
35	26	10	8	69.23
Minimum	3	1	1	
Maximum	32	21	15	
Average	16.29	9.26	5.17	68.26

The repeated measures analysis of variance confirmed that there was a significant change in scores during the evaluation times (Repeated measures ANOVA: *F*_(6,204)_ = 69.95, *P* < 0.001). Significantly higher values were observed for W0 than for the other periods (Repeated measures ANOVA contrast W0-W2: *F*_(1,34)_ = 4.24, *P* = 0.047; contrast W0-W4: *F*_(1,34)_ = 38.52, *P* < 0.001; contrast W0-W6: *F*_(1,34)_ = 63.56, *P* < 0.001; contrast W0-W8: *F*_(1,34)_ = 88.75, *P* < 0.001; contrast W0-W10: *F*_(1,34)_ = 88.82, *P* < 0.001; contrast W0-W12: *F*_(1,34)_ = 107.02, *P* < 0.001). Significantly higher values were also observed for W6 than for W12 (Repeated measures ANOVA: *F*_(1,34)_ = 44.87, *P* < 0.001).

### Skin qPCR

At W0, an average of 130,988.5 amplified copies was estimated in the qPCR, but six weeks after beginning the treatment (W6) a drastic reduction in parasitic load was detected (99.8%). At W12, the reduction was 98.7%, as shown in Table 3.

**Table 3:** Parasite load (number of amplifications) in skin samples determined by qPCR

Dog ID	DNA amplification			Log_10_		
	W0	W6	W12	W0	W6	W12
1	0	0	0	−	−	−
2	0	3.27	0	−	−	−
3	0	0	0	−	−	−
4	0	0	0	−	−	−
5	0	0	0	−	−	−
6	125.73	0	0	2.10	−	−
7	1230.63	177.68	769.60	3.09	2.25	2.89
8	2.94	0	0	0.47	−	−
9	364,348.05	0	1.65	5.56	−	0.22
10	316.23	0	0	2.50	−	−
11	105.76	7.1	12.43	2.02	0.85	1.09
12	0	0	1450.97	−	−	3.16
13	1.64	0	0	0.21	−	−
14	225.61	0	2.17	2.35	−	0.34
15	67.24	0	0	1.83	−	−
16	2946.06	0	10.12	3.47	−	1.01
17	0	0	0	−	−	−
18	1,685,592.16	343.37	93.47	6.23	2.54	1.97
19	263.83	3.25	132.10	2.42	0.51	2.12
20	3172.71	3710.09	55,727.02	3.50	3.57	4.75
21	0	0	0	−	−	−
22	7658.08	465.69	454.33	3.88	2.67	2.66
23	26.51	0	0	1.42	−	−
24	0	0	0	−	−	−
25	42,817.04	0.10	421.87	4.63	-1	2.63
26	77,468.89	85.37	68.35	4.89	1.93	1.83
27	0	0	0	−	−	−
28	923,985.67	4.48	30.50	5.97	0.65	1.48
29	3.80	0	0	0.58	−	−
30	601.13	1.31	0	2.78	0.11	−
31	324.14	0	0	2.51	−	−
32	0	1.30	4.37	−	0.11	0.64
33	13.50	0	0	1.13	−	−
34	7914.55	0	0	3.90	−	−
35	1,465,383.71	9.63	277.19	6.17	0.98	2.44
Average	130,988.45	137.50	1698.75	3.07	0.43	1.95

The repeated measures analysis of variance showed a significant change in qPCR results during the evaluation period (Repeated measures ANOVA: *F*_(2,68)_ = 15.20, *P* < 0.001). Week 0 showed significantly lower values than the other periods (Repeated Measures ANOVA contrast W0-W6: *F*_(1,34)_ = 19.32, *P* < 0.001; contrast W0-W12: *F*_(1,34)_ = 17.01, *P* < 0.001) and W6 did not show any difference in comparison to W12 (Repeated measures ANOVA contrast W6-W12: *F*_(1,34)_ = 0.84, *P* = 0.366).

### Xenodiagnosis

In the xenodiagnosis at W0, 785 female sand flies, with an average of 22.4 females per dog, were dissected. *Leishmania* promastigotes were detected in 82 of them (Fig. 1j). Of the 35 dogs included in the study, 18 were infective to the sand flies at W0. At W12 only 9 dogs were infective to the sand flies. At the end of the observation period, 74.29% of the dogs were negative and/or remained non-infective (Table 4).

**Table 4:** Sand flies dissected and infected with *Leishmania* spp.

Dog ID	Positive phlebotomines/total dissected		Percentage (%)	
	W0	W12	W0	W12
1	0/20	0/25	0	0
2	0/40	0/25	0	0
3	0/21	0/25	0	0
4	0/20	0/25	0	0
5	0/26	0/25	0	0
6	0/25	0/25	0	0
7	2/33	0/25	6.06	0
8	0/39	0/25	0	0
9	3/24	1/21	12.50	4.76
10	1/15	0/25	6.67	0
11	0/28	0/25	0	0
12	0/25	1/20	0	5.00
13	0/35	0/25	0	0
14	3/20	0/25	15.00	0
15	1/25	0/25	4.00	0
16	2/16	0/25	12.50	0
17	0/17	0/18	0	0
18	19/20	13/20	95.00	65.00
19	1/20	0/25	5.00	0
20	0/20	14/20	0	70.00
21	0/25	0/25	0	0
22	3/21	5/20	14.29	25.00
23	1/20	0/25	5.00	0
24	0/25	0/16	0	0
25	1/21	5/20	4.76	25.00
26	13/20	7/20	65.00	35.00
27	0/22	0/21	0	0
28	6/10	7/20	60.00	35.00
29	1/20	0/25	5.00	0
30	0/20	0/25	0	0
31	0/11	0/15	0	0
32	4/21	0/25	19.05	0
33	2/20	2/20	10.00	10.00
34	4/20	0/25	20.00	0
35	15/20	0/25	75.00	0
Total	82/785	55/801		

## Discussion

In the present study a progressive and statistically significant decrease in clinical scores was observed and verified after administration of miltefosine. Of the 35 dogs evaluated, 33 (94.28%) showed clinical improvement; the mean score reduction was 68.26% at week 12, and only two animals with low scores at week 0 (W0) showed no improvement. The reduction or absence of clinical signs could decrease infectivity to sand flies according to various authors [40–42].

Our results are in accordance with other studies using miltefosine for the treatment of VL sick dogs. Treating naturally infected dogs with only miltefosine [18], a reduction in clinical scores was observed, resulting in a 61.2% mean on day 56. Similarly, using a therapy combining miltefosine and allopurinol, Miró et al. [16] observed a significant reduction in clinical scores and parasite load, providing evidence that miltefosine treatment of infected and sick dogs produces a significant clinical improvement in those animals.

Regarding *L. infantum* DNA detection by qPCR, a drastic decrease was observed at six and 12 weeks after initiation of therapy. These results suggest that miltefosine could reduce parasite load in the skin of treated dogs. Duration of the treatment was 28 days and the reduction in parasite DNA occurred at least until week 6, indicating a continuity of the drug’s effect.

The results obtained in our study also demonstrate that qPCR is an important tool for the detection of *Leishmania* DNA in tissues, mainly in the skin, given its high diagnostic sensitivity, as previously pointed out by other authors [31, 40–43]. It is known that skin is an important tissue in CVL diagnostics because of its high parasitism and as a source of infection [44]. Therefore, these data indicate that the use of qPCR to detect parasite DNA in skin could be an important tool for detection of infected but clinically healthy dogs in endemic areas due to its practicality, accuracy and ease of use.

Regarding the infectiveness of the dogs in our study, the results showed a reduction in the number of dogs that were infective to sand flies. The results reinforce our hypothesis that treated dogs are less infective to sand flies, as previously suggested by other authors [42]. Undoubtedly, the treatment of infected dogs does not result in parasitological cure [3, 16, 17, 43, 45]; however, our results suggest that therapy with miltefosine contributes to a reduction in the infectiveness of treated dogs.

The general improvement observed is supported by the clinical scores and results on parasitic load in the skin obtained by qPCR. Considering the different methods to evaluate the treated dogs (clinical scores, qPCR and xenodiagnosis), the present study observes a similarity between the results of xenodiagnosis and those of qPCR.

Our study demonstrated that the use of miltefosine showed potential for reducing the parasitic load of dogs infected with *L. infantum*; clinical improvement in the dogs was also observed. These results are in agreement with the observations by Woerly et al. [18], showing a reduction in clinical scores, and the observations of Andrade et al. [46] observations of the progressive clinical improvement and recovery of 50% of the dogs during the 24 months of the study, although it was not clear to which of the three treatment groups they belonged.

Our data also proved the safety of miltefosine, considering that none of the dogs experienced vomiting or other adverse reactions to the drug at any time during the study period (28 days). This was similar to results obtained by Miró et al. [16], but different from the observations of Woerly et al. [18] showing that 11.7% of the dogs had adverse reactions.

The results presented herein demonstrate the potential of miltefosine as an alternative treatment for infected dogs in regions where this drug is not used in humans. It should be pointed out that previous studies demonstrated that better results have been obtained with complementary/combined therapies [3, 16, 17, 31, 41, 47]. It is mandatory to carefully observe the treated dogs for the rest of their lives to avoid any possibility of drug resistance.

Considering that the treatment does not cause parasitological cure in treated dogs, it is important to stress the importance of adopting preventive measures for protection of individuals under treatment [45], such as the use of repellents and insecticides to diminish their contact with the vector, as well as measures of environmental control aimed at reducing the vector population.

## Conclusions

In conclusion, we observed that the use of miltefosine administered orally for 4 weeks contributed to a clinical improvement and reduction in infectivity of dogs to *L. infantum.* Agreement was observed between clinical scores and results obtained by xenodiagnosis and by skin qPCR. There was a statistically significant reduction in parasite load, as evidenced by qPCR from skin. In addition, xenodiagnosis demonstrated a reduction in infectivity of the dogs to sand flies, during the 90 day observation period. These results contribute by offering an important measure to complement the control programs of visceral leishmaniasis in transmission areas of Brazil.

## Abbreviations

BEPA: Boletim Epidemiológico Paulista
CDC: Center for Disease Control
CVL: Canine visceral leishmaniasis
DPP: Dual path platform
ELISA: Enzyme-linked immunosorbent assay
PCR: Polymerase chain reaction
qPCR: Real-time polymerase chain reaction
VL: Visceral leishmaniasis
WHO: World Health Organization
